# On Dam-Break Flow Routing in Confluent Channels

**DOI:** 10.3390/ijerph16224384

**Published:** 2019-11-09

**Authors:** Sihan Chen, Yingjin Li, Zhong Tian, Qiang Fan

**Affiliations:** 1 State Key Laboratory of Hydraulics and Mountain River Engineering, Sichuan University, Chengdu 610065, China; 2 Yunnan Institute of Water & Hydropower Engineering Investigation, Design and Research, Kunming 650021, China

**Keywords:** dam-break flood, physical testing, confluent channel, numerical simulation, retardation, abatement

## Abstract

The flood propagation at a confluence of channels exhibits a unique routing pattern, while there are few studies on the routing of dam-break flow in confluent channels. In this study, we conducted physical experiments and a numerical simulation to investigate the influence of different confluence angles on the routing of a dam-break flood. Experiments were carried out in smooth, transparent, rectangular prismatic channels to study the dam-break flow under four different confluence angles. The flow velocity was measured using an image processing technique, and the surface flow field was effectively captured by synchronously recording the particle motion images. Based on the variation of the water level and flow discharge, as the confluence angle increased, the retardation and abatement effects on the flood increased. Specifically, the flood arrival time was delayed by approximately 0.91% to 21.18%, and the peak flood discharge was reduced by approximately 9.05% to 58.36%. Combined with the surface flow field at the confluence and in the downstream sections, as the confluence angle increased, the impact points at the confluence and in the downstream straight sections moved upward, and the impact range was reduced. Combined with the pressure variation pattern, the routing of dam-break flow in the confluent channels experienced a process of impact-reflection-return-attenuation.

## 1. Introduction

River confluence is commonly found in natural rivers, with complex flow conditions at the confluence area. When a hydroelectric dam constructed upstream of a river breaks under a dangerous situation, the large amount of water stored in the reservoir forms a dam-break flood whose routing along the natural courses of the river leads to catastrophic damage to towns and infrastructure on both sides of the river. Different from ordinary floods, the dam-breaking floods happen very suddenly and quickly, and the wave peak value and peak flow discharge are several times that of rainstorm floods. When the dam-break flow propagates downstream, the velocity is extremely high and the flow is disorderly, seriously impacting the normal activities of the local society and economy. Due to the complex boundary conditions of the river confluence area, the hydraulic factors such as the flow field and water depth of a dam-break flood vary in this area, resulting in unique hydraulic phenomena [1,2] such as secondary flow, spiral flow, local flow stagnation, separation, and water superelevation, thereby leading to a routing pattern that is very different from that in a single river channel.

Based on the complexity of dam-break flood propagation and the severity of its consequences, some researchers have carried out a series of studies on the hydraulic characteristics of dam-break flood routing and flood propagation in confluence channel to reduce the risk of dam-break floods. Lauber and Hager [3,4] conducted a dam-break wave experiment in a horizontal channel to investigate the effects of flow velocity distribution, boundary roughness, and cross-sectional reduction on the routing of dam-break flow. Marry et al. [5] captured motion characteristics such as the water surface profile and wavefront velocity of the dam-break flow under dry bed conditions with various bed slopes and analyzed the effects of bed slope angle on the motion parameters. H. von Hafen and G. Goseberg [6] examined the influence of the gate opening on characteristics of the wave propagation by Smoothed Particle Hydrodynamics (SPH). Soares-Frazao and Y. Zech [7] used an image acquisition method to obtain the variation in the water depths of dam-break floods in rectangular channels with 90° bends and combined a numerical simulation method to study the propagation pattern. Fraccarollo and Tora [8] established a shallow-water model for the problem of a two-dimensional (2D) dam-break on a dry bed and verified the model through laboratory tests. Macchione et al. [9] compared the practical aspects of the first order central difference and the second-order total variation diminishing (TVD) scheme in the calculation of dam-break waves. Hu et al. [10] compared results from Flow 3D and Mike 3FM models and analytical solutions with experimental data, showing that Flow 3D captures the wavefront free surface profiles of dry and wet beds better; Alex Ghaitanelis et al. [11] tested the model on a case of a dam-break wave propagating a saturated bed and then found the dynamics of flow were qualitatively well reproduced by the model, established by Smoothed Particle Hydroynamics (SPH) multi-phase formulation combined with a granular rheological model. Liu et al. [12,13] studied the three-dimensionality of the water flow in the confluence area and the proximity of the flow with tributary angles of 30° and 90° using acoustic doppler velocimetry (ADV). Chow [14] proposed an unsteady flow model that can accurately handle the confluence of tributaries. Shi et al. [15] conducted numerical simulation of the water depth of the branching channels along the river under different confluence angles and different widths using the generalized Boussinesq long wave model and carried out T-shaped branching channel flow tests to investigate the mechanisms and patterns of the propagation of long water waves through branching channels. Marsooli [16] used the finite volume method to analyze and calculate three-dimensional (3D) dam-break flood flow and satisfactorily capture the free water surface over uneven beds. Peng [17] used Multispeed Lattice Boltzmann Model (LBM) to calculate high Froude number (Fr) dam-break flow and satisfy accuracy. The rapid development of computer and image vision technology has provided new tools for research. Eatek et al. [18] used three cameras to simultaneously capture tracer particles spread over the water surface and obtained the velocity distribution of a dam-break flood by analyzing the particle motion between consecutive photographs. Aleixo et al. [19] used a particle image velocimetry (PIV) technique to measure the cross-sectional velocity of the one-dimensional dam-break flow and obtain the cross-sectional flow velocity distribution. With social intelligence, Omid Seyedashraf [20] proposes a new method based on a computational intelligence (CI) system, and based on the best fitting model, a new global equation is proposed, which can be used to directly solve dam-break problems. S. Sina Hosseini Boosari [21] uses artificial intelligence (cascade deployment) to predict the dynamic parameters of multiphase flow in CFD (dam-break simulation) under the condition of ensuring accuracy, and greatly shortens the time. Other researchers have conducted related studies [22,23].

A summary of the previous research results reveals that most researchers have focused on the routing of dam-break floods in straight channels, channels with bends, or channels with obstructions [24,25,26], emphasizing the hydraulic characteristics of the natural channels at the confluence of the main stream and tributaries [27,28], while there are few studies on the routing of dam-break flow in confluent channels. In the current study, the characteristics of dam-break flood flow in confluent channels were analyzed, the variations and distributions of pressure in the confluent channels and on the downstream sidewalls with time were studied through physical model tests, the distributions of the surface flow fields in the confluent and downstream sections were measured based on PIV principles, and the water level at each point along the river was compared with that of the straight channel to analyze the influence of different confluence angles on the routing of dam-break floods. Furthermore, the characteristics of dam-break floods in confluent channels were simulated using FLOW-3D software to obtain the variation in dam-break flood flow under different confluence angles. The relevant results can provide data support for future theoretical analyses and are very significant to the validation of numerical calculations and preparation plans for dam-break flood prevention.

## 2. Materials and Methods

### 2.1. Physical Model

The current research on dam-breaks is mainly based on numerical simulation and statistical analysis of historical data, but physical model test is still an indispensable technical means to study the dam-break. The dam-break model experimental results not only make up for the limitation of the amount and reliability of the dam-break history data, but also provide verification data for numerical simulation, and it has unparalleled reliability in dam-break flood routing.

#### 2.1.1. Arrangement of Test Apparatus

**Figure 1:**
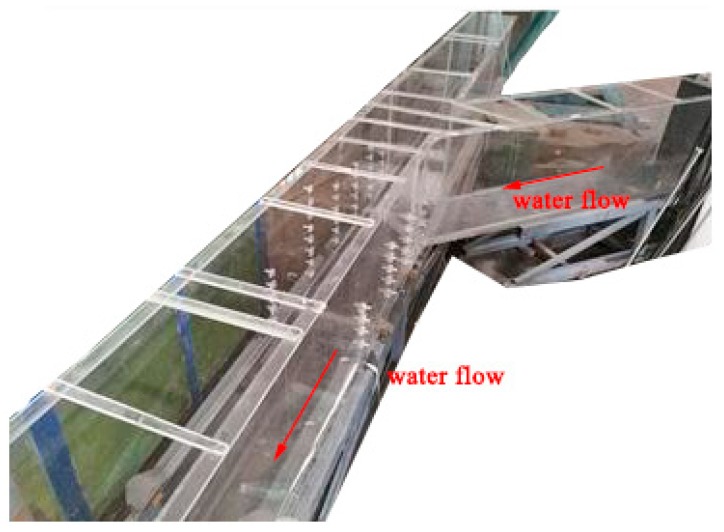
Photograph of the test apparatus.

**Figure 2:**
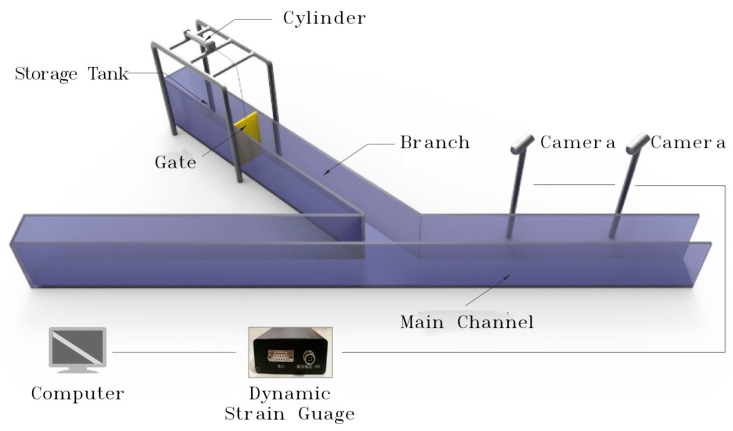
Sketch of the dam-break model test systems.

**Figure 3:**
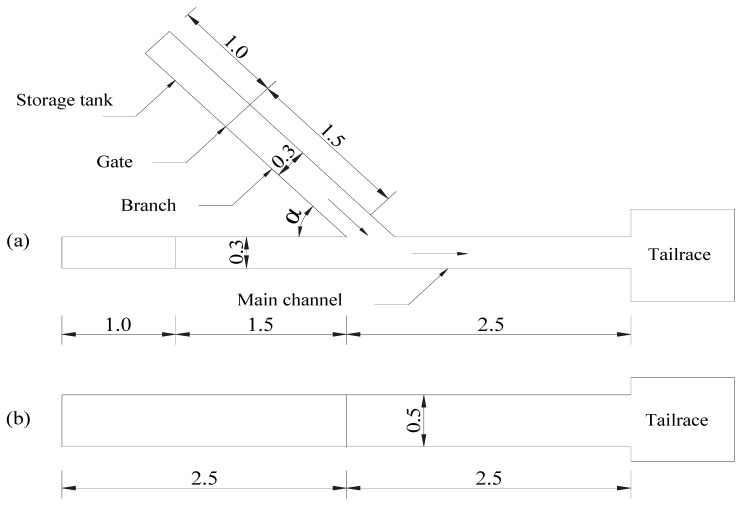
Sketch of the test channel, (**a**) plan view; (**b**) elevation (unit: m).

As the shown in the Figure 1, Figure 2 and Figure 3, this experiment ensured that the image acquisition effect was appropriate for observing the characteristics of the water flow conveniently. Involved devices were made of transparent plexiglass (coefficient of rugosity n = 0.008), and the whole experimental system was composed of four parts: water channel, gate, water storage equipment, and downstream backwater area. The test tank consisted of a flat-bottomed prism branch and main channel, in which the main channel was divided into two parts, the downstream of the intersection was 2.5 m, and the upstream was 1.5 m. In addition, the length of the branch was 1.5 m, the width of the water channel *b* was 0.3 m, the height of *h* was 0.5 m, and the water reservoir length was 1.0 m arranged in the upstream of the branch and main channel. Meanwhile, the backwater system was also set up downstream of the tank.

A gate was set 1.5 m from the confluence of the main and branch channels, and a fixed pulley was installed directly above the gate. In this test, an instantaneous dam-break was simulated by a weight falling freely through the pulley to instantaneously lift the gate. The time needed for the gate to rise from the bottom to the top should satisfy Equation (1) which was proposed by Lauber [4] and Hager to determine whether the dam-break instantaneously collapsed or not.

(1) $$t<\sqrt{\frac{2h_{0}}{g}},$$

where *h*_0_ in the upper formula is the initial water depth of upstream of the dam-break, *g* is the acceleration of gravity, 9.8 m^3^/s. It was calculated that the dam-break duration of different water depths in the upstream of the different vents in this test was 0.19 and 0.27 s, respectively. It is obvious that the experimental results are less than the time of 0.25 and 0.30 s, calculated by the formula, so the rising speed of the gate meets an instantaneous dam-break condition.

#### 2.1.2. Test Methods

Dam-break flow is an unsteady, rapidly varying flow, that is, a flow that drastically changes over a short period of time. Conventional measurement methods (such as current meters and Pitot tubes) are unable to accurately and effectively obtain dam-break flow velocity. Therefore, based on PIV principles, an image processing method was used in the current experiment for flow velocity measurements. This method does not need to be in contact with the flow, thereby avoiding interfering with the flood propagation, and can accurately reflect the flow regime. A pulley track was installed directly above the channels to change the position of the industrial camera. The flow velocity measuring system included tracer particles (polypropylene, with a density of approximately 0.92 g/cm^3^ and a particle length of 4 mm), two industrial cameras, a synchronous controller, and a computer with image acquisition software. The flow velocity was measured using three steps. In the first step, the industrial cameras were calibrated, which placed the ruler in the model, the direction was parallel to the width and length of the sink to determine their range. Then, when compared to the camera resolution, the calculated coordinates were transformed to 1 pixel = 0.00047 m and 1 pixel/frame = 0.09375 m/s in the second step, with the synchronous controller, the images of the dam-break flow were transmitted to the computer. The raw images recorded were digitized as 640 × 480 pixels at 200 frames/s. In the last step, each particle motion image acquired during two adjacent time points was processed in MATLAB to obtain the flow velocity distribution. Additionally, since measuring the variation in the water level is difficult, the water depth was measured using an SDA1000 high-performance digital sensor system that had a sampling frequency as high as 100 Hz, a high-performance single-chip microcomputer system, and a high-precision AD collector as the core.

#### 2.1.3. Test Conditions and Placement of Measuring Points

In the current experiment, only the confluence angle of the main and branch channels was changed, while the other dimensions and the boundary conditions remained the same, resulting in four configurations each with a different confluence angle. In addition, for comparison purposes, a straight channel with a total length of 5 m was added. For each configuration, the upstream water level *h*_0_ was set to 0.30, 0.40, and 0.45 m. Table 1 lists all the test conditions.

**Figure 4:**
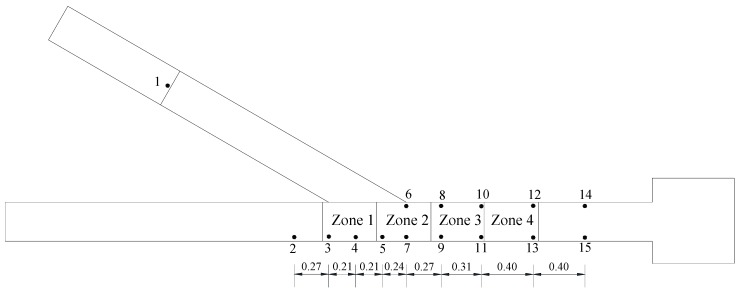
Layout of measuring points in the tests (unit: m).

As shown in Figure 4, a total of 15 water level measuring points were set along the channel, of which nine measuring points were selected for pressure measurements. A total of four flow velocity measurement areas were used to study the water level, pressure, and flow field at the confluence and downstream of the dam-break flood. To facilitate the comparison, the water depth in the test was non-dimensionalized by using H = *h*/*h_0_*, which is the ratio of the measured water depth *h* to the upstream initial water depth *h*_0_, to represent the water depth at the measuring point and by using L = *l*/*b*, the ratio of *l*, which is the distance from the measuring point to the confluence O, to *b*, which is the width of the channel, to represent the locations of the measuring points and cross-sections.

#### 2.1.4. Verification of Test Repeatability

**Figure 5:**
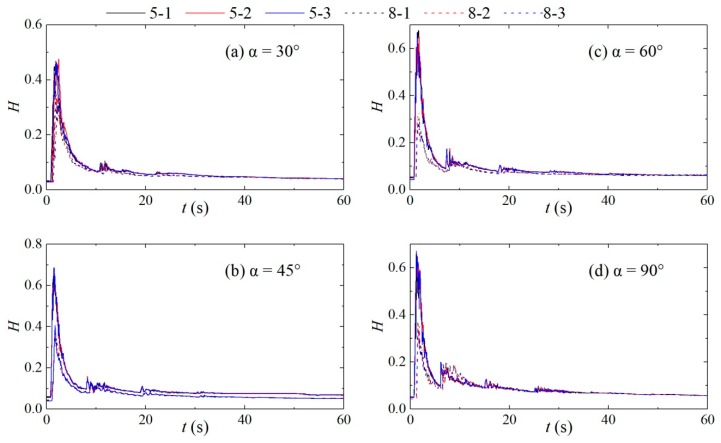
Variations in the water depth at measuring points #5 and #8. (**a**): α = 30°; (**b**): α = 45°; (**c**): α = 60°; (**d**): α = 90°.

To verify the reliability and validity of the data, each test condition was tested three times. Taking the water depth of 0.30 m upstream of the dam-break as an example, the test data from the measuring points #5 and #8 with different confluence angles were selected for analysis. Figure 5 shows the variation in the water depth at each measuring point. Clearly, for the same confluence angle and the same upstream water level, the variations in the water depth at the same measuring point for the three tests basically coincide with each other. Since the dam-break flow is an unsteady, rapidly varying flow, the impact and reflection occur at the junction, causing relatively large fluctuations, 10% can be defined as the maximum allowable error. The difference between the peaks in this experimental result is 0.44–8.59%, within the experimental error range. Therefore, there was good repeatability in this experiment.

### 2.2. Numerical Model

The physical test of the dam-break flow can provide technical parameters for the design of the actual project, but it is difficult to obtain the distribution of the flow field inside the dam-break flow. While, the FLOW-3D numerical model can supplement the physical model test and comprehensively explore the hydraulic characteristics inside the dam-break flow.

#### 2.2.1. Turbulence Model and Free Surface

Five turbulence models, namely, the standard *k*-*ε* model, RNG *k*-*ε* model, one-equation model, Prandtl mixing-length model, and large eddy simulation (LES), are provided by FLOW-3D. Larocque [29,30] used the LES and the *k*-*ε* turbulence models to investigate 2D and 3D dam-break flow problems, compared the data with experimental results, and concluded that the LES model can capture the details of the elements of dam-break flow motion better. Therefore, the LES model was used for simulation in the current study.

The main principle of the LES is to perform direct numerical simulation only for eddy motions larger than the grid scale, while eddy motions smaller than the grid are calculated using an empirical closed model based on an isotropic assumption. Large and small eddies are differentiated in the LES through a weighted integral in the physical space domain. The expression is

(2) $$\bar{f}(x,t)=\int (x-x^{\prime })f(x^{\prime },t)d\sigma ,$$

where *G (x-x′)* is the weight function. A filter operation is applied to the N-S equation of instantaneous motion to obtain the governing turbulence equations in the LES

(3) $$\frac{\partial (\rho \bar{u_{i}})}{\partial t}+\frac{\partial (\rho \bar{u_{i}u_{j}})}{\partial x_{j}}=\frac{\partial }{\partial x_{j}}(\mu \frac{\partial u_{i}}{x_{j}})-\frac{\partial \bar{p}}{\partial x_{j}}-\frac{\partial \tau_{ij}}{\partial x_{j}},$$

(4) $$\frac{\partial \rho }{\partial t}+u\frac{\rho \bar{u_{i}}}{\partial x_{i}}=0,$$

where *τ_ij_* is the subgrid-scale stress, which is calculated by the currently widely used Smagorinsky model.

The Tru-Volume of fluid (VOF) method is a unique free surface tracking technique in Flow-3D. In 1981, Hirt and Nichols [31] proposed a VOF method to simulate dam-break flow; the free surface was accurately simulated, and the concept of fluid volume function was introduced. The core idea of the Tru-VOF method is as follows: the computational units are all composed of water and gas; the fluid volume function F is the ratio of the volume of liquid to the unit volume in a computational unit; when F = 0, the unit is all gas; when F = 1, the unit is all liquid; and when 0 < F < 1, the unit is a mixture of gas and liquid and contains a free surface. In Flow-3D, the free surface is tracked using the following continuity equation:

(5) $$\frac{\partial F}{\partial t}+u_{i}\frac{\partial F}{\partial x_{i}}=0,$$

where *u_i_* and *x_i_* are the velocity component and coordinate component, respectively, and *t* is time.

#### 2.2.2. Computational Domain, Boundary Conditions, and Initial Conditions

The computational domain of the numerical model is consistent with the physical experimental model device and has a grid size of 0.02 (X-direction) × 0.02 (Y-direction) × 0.01 m (Z-direction). The RNG *k*-*ε* turbulence model is used in the calculation, and a gravity model is set up with an acceleration of −9.81 m/s^2^. The general moving object (GMO) module is used to simulate gate motion. Based on the physical tests, rugosity of channel is 0.008, and the gate-lifting speeds at the initial upstream water depths of 0.30 and 0.45 m are set to 2.632 and 1.852 m/s, respectively. To measure the variation in the flow with time at specified cross-sections, a total of six baffles were set up at cross-sections of the confluence and downstream. The cross-section locations are shown in Table 2.

The boundary conditions for the upper part of the water channel were set as a pressure boundary, and the pressure was set as the atmospheric pressure. The outlet of the water channel was set as a free discharge, the upstream end and the bottom of the water channel were both set as solid-wall boundaries, and the sidewalls of the water channel were set as symmetrical boundaries. The body of water upstream of the gate was initially static, and the initial water depth was set according to the corresponding test conditions.

#### 2.2.3. Reliability Analysis of Numerical Simulation Results

**Figure 6:**
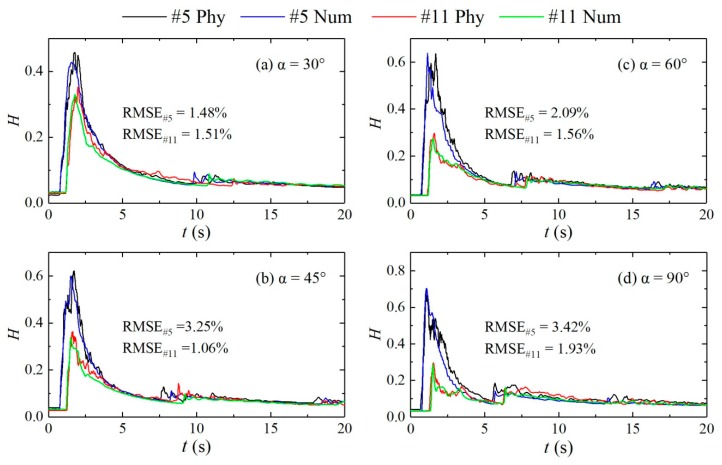
Comparisons of water depths at measuring points #4 and #10 from numerical simulation and experiment. (**a**): α = 30°; (**b**): α = 45°; (**c**): α = 60°; (**d**): α = 90°.

To verify the accuracy of the numerical simulation results, the results were compared with the results from the physical experiment. As an example, the numerical simulation results for the water levels at measuring points #4 at the confluence and #10 in the downstream section for different configurations with an initial water depth of 0.30 m upstream of the dam-break and within a duration of 20 s of the dam-break were compared. As shown in Figure 6, the numerical simulation results agree well with the physical test results. The variations in the water depth with time are basically consistent. The root mean squared error (RMSE) (Equation (6)) between the results of the physical tests and numerical simulation of the #4 measuring point is changed to 1.48%, 2.09%, 3.25%, 3.42%, and the RMSE of the #11 measuring point is 1.51%, 1.56%, 1.06%, and 1.93%, respectively. The values are all small, it can be seen that the numerical simulation results are in good agreement with the physical model experiment, and the maximum difference of 5.68% in the wave peak values is within the allowable range of the experimental measurement error. The 2D surface flow fields obtained by numerical simulation and experiment are qualitatively compared in Figure 6. The results show that the flow regimes at the confluence and in the downstream straight section obtained by numerical simulation are consistent with those of the physical model test results, with maximum differences of 5.56% and 8.69% for the flow velocities at the inlet and in the downstream straight section, respectively. Therefore, the numerical simulation results are reasonable and can simulate the routing of dam-break floods well.

(6) $$RMSE=\sqrt{\frac{\sum_{m=1}^{n}(h_{Phy}-h_{Num}{)}^{2}}{n}},$$

In the formula, *h_Phy_* is the physical test water depth, *h_Num_* is the numerical simulation water depth, and *n* is the number of samples.

## 3. Results and Discussion

### 3.1. Water Level Variation

**Figure 7:**
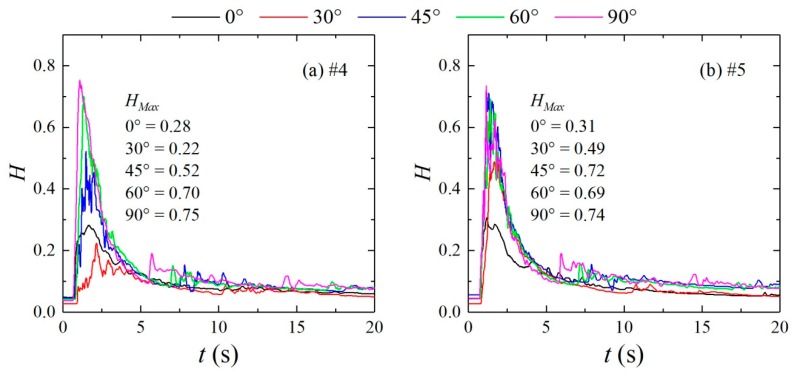
Variations in the water depths of different configurations at measuring points #4 and #5 over time. (**a**): measuring points #4; (**b**): measuring points #5.

Figure 7 compares the variations in the water depth at measuring points #4 and #5 for an initial upstream water depth of 0.30 m. The figure shows that the dam-break flow quickly reaches a peak at approximately 0.76 s. However, at this time, the flow regime is turbulent, and the depth fluctuates as water level decreases; after the wave peak is reached, the relative water depth fluctuates from 6 to 10 s and then stabilizes. The wave peaks at measuring points #4 and #5 differ for different confluence angles. As the confluence angle increases, the water depth of the wave peak increases. This phenomenon is due to the obstruction effect of the right bank of the confluence section on the dam-break flow, causing the water level to increase at the impact of flow on the right bank. As a result, the wave peak is larger than that at the same position in the straight channel, and as the confluence angle increases, the water level and the wave peak increase. A comparison of the water depths of the wave peaks at measuring points #4 and #5 shows that the water depth of the wave peak at measuring point #5 is greater than that of measuring point #4 at confluence angles of 30° and 45°, and the water depth of the wave peak at measuring point #4 is greater than that of measuring point #5 at confluence angles of 60° and 90°. This phenomenon occurs because the location impacted on the right bank differs with the confluence angle. When the confluence angle increases, the impact point moves upstream, and the wave peak of the impact point increases.

**Figure 8:**
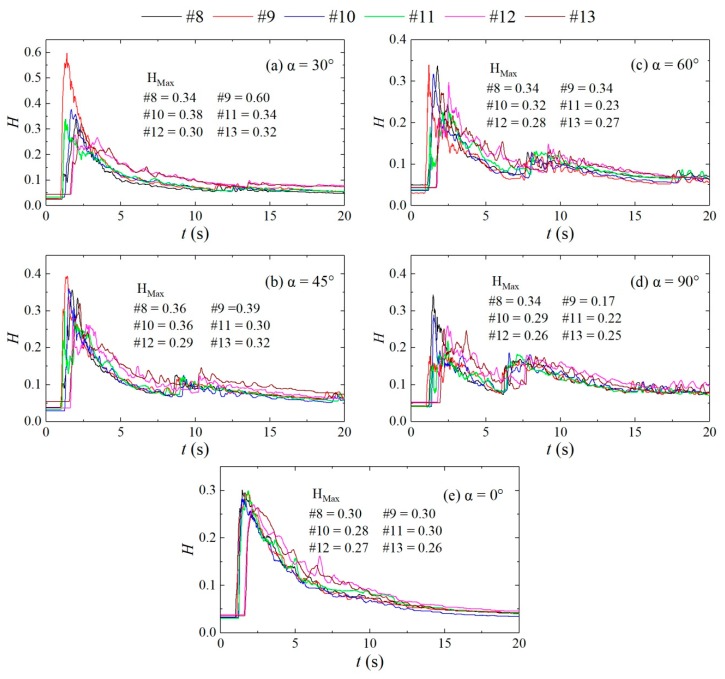
Variations in the water depths of the downstream straight sections of different configurations with time. (**a**): α = 30°; (**b**): α = 45°; (**c**): α = 60°; (**d**): α = 90°; (**e**): α = 0°.

Water superelevation occurs at the downstream straight section due to the influence of the confluence, and the flow exhibits notable asymmetry. As shown in Figure 8, in the straight channel, the water depths at the measuring points on the left and right banks of the cross-sections L = 3.1, 4.1, and 5.4 are basically consistent, while the wave peaks at measuring point #8 in configurations with different confluence angles do not significantly differ, and the wave peaks are all larger than those at the same location in the straight channel. The wave peak at measuring points #9, #10, #11, #12, and #13 decrease with increasing the confluence angle. Additionally, compared with the values at the same locations in the straight channel, the relative values differ with different confluence angles. Under confluence angles α = 60° and 90°, the wave peaks at measuring points #11, #12, and #13 are smaller than those in the straight channel, and the opposite is true under other confluence angles. Under different confluence angles, the water superelevations at cross-sections L = 3.1, 4.1, and 5.4 differ. Under α = 30°, the water level at the right bank is significantly higher than that at the left bank at cross-section L = 3.1; under α = 45° and 60°, two superelevations occur, and when the dam-break flow reaches the cross-section L = 3.1, the water level at measuring point #9 rapidly rises to the wave peak and then declines, while the water level at measuring point #8 rises slowly, causing the left bank water level to be first lower and then higher than the right bank water level; under α = 90°, the situation is opposite that under α = 30°, that is, the water depth at the left bank is significantly higher than that at the right bank. At the cross-section L = 4.1, the water level at the left bank is higher than that at the right bank under different confluence angles, and at the cross-section L = 5.4, the difference between the water levels of the left and right banks is relatively small; as the confluence angle increases, the water superelevation phenomenon becomes less obvious.

**Figure 9:**
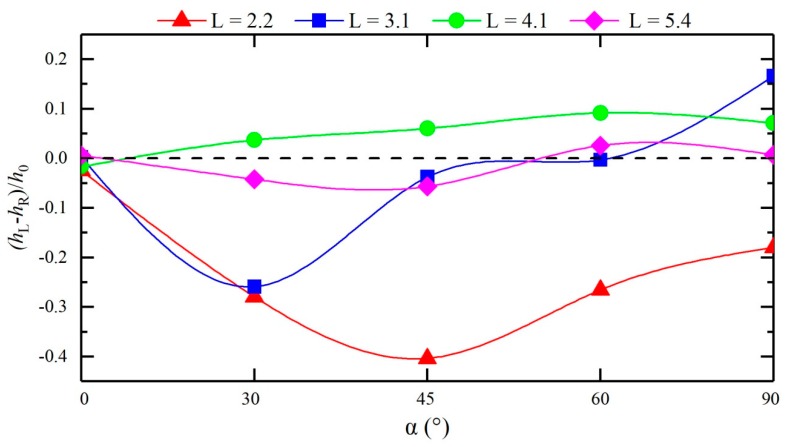
Comparison of the difference between the wave peaks at the left and right banks at each cross-section of the downstream straight section of each configuration.

Figure 9 compares the difference between the peaks on the left and right banks of the cross-sections in the downstream straight section under different confluence angles. Clearly, as the distance to the downstream decreases, the difference between the peaks of the left and right banks under different confluence angles decreases. Combined with the water superelevations at the cross-sections L = 3.1, 4.1, and 5.4, the ranges of the water superelevation in the downstream straight sections differ under different confluence angles; as the confluence angle increases, the range of the water superelevation first decreases and then increases; in particular, the range of the water superelevation is the smallest under a confluence angle of 60°.

**Figure 10:**
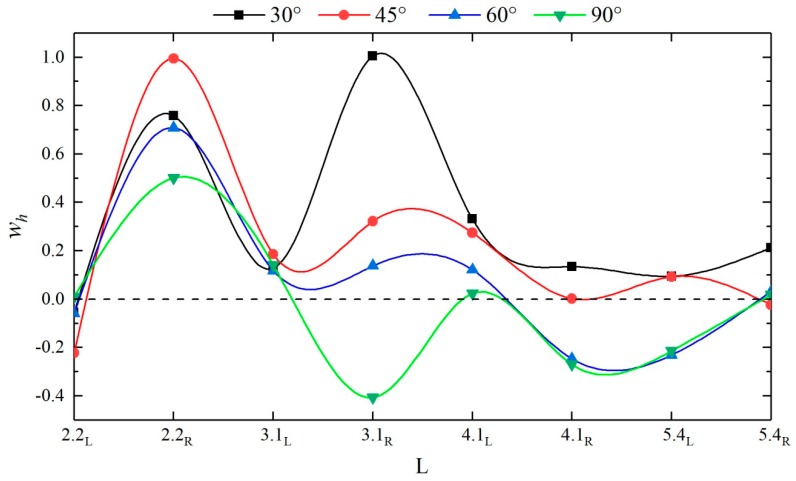
*w_h_* values at each measuring point in the downstream straight sections of different configurations.

The above discussion shows that different confluence angles lead to different wave peaks on the two sides of the same cross-section, and under different confluence angles, the sizes of the wave peaks at the two sides differ. A wave peak factor *w_h_* is introduced to analyze the influences of different confluence angles on the wave peaks of different downstream measuring points by Equation (7).

(7) $$w_{h}=\frac{h_{j,\max}-h_{s,\max}}{h_{s,\max}},$$

where *h_j_* is the wave peak at a measuring point in the confluence channel and *h_s_* is the wave peak at the same measuring point in the straight channel. Figure 10 shows the values of the wave peak factor at each measuring point in the downstream straight sections of different configurations. A *w_h_* greater than 0 indicates that the confluence angle enhances the wave peak at this measuring point and vice versa. A comparison reveals that due to the existence of a flow separation region at the confluence, the wave peaks at measuring point #6 are all reduced, while at 30°, 45°, and 60°, the dam-break flow impacts the right bank at the confluence, and the wave peaks at measuring points #7 and #9 both show large increases; as an overall trend, the curve as a whole starts to fluctuate. At L = 4.1, the curve stars to move closer to 0, that is, after the dam-break flow impacts the right bank at the confluence, the flow then propagates downstream in broken lines, and the flow starts to gently propagate downstream at L = 4.1; the confluence angles increase the wave peak at the measuring points by approximately 0.19% to 99.49%, and the smaller the confluence angle is, the more remarkable the enhancement effect is; when the confluence angle decreases the wave peak, this decrease ranges from 1.82% to 40.70%, and the larger the confluence angle is, the greater the decrease is.

**Figure 11:**
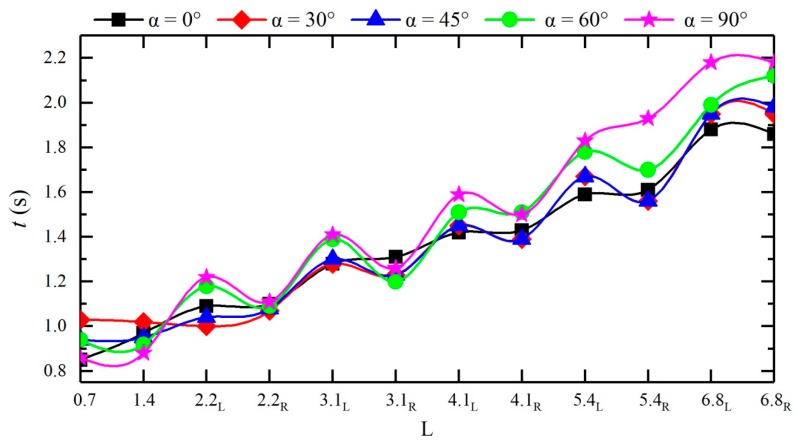
Water depth rise time *t_j_* at each measuring point of different configurations.

**Figure 12:**
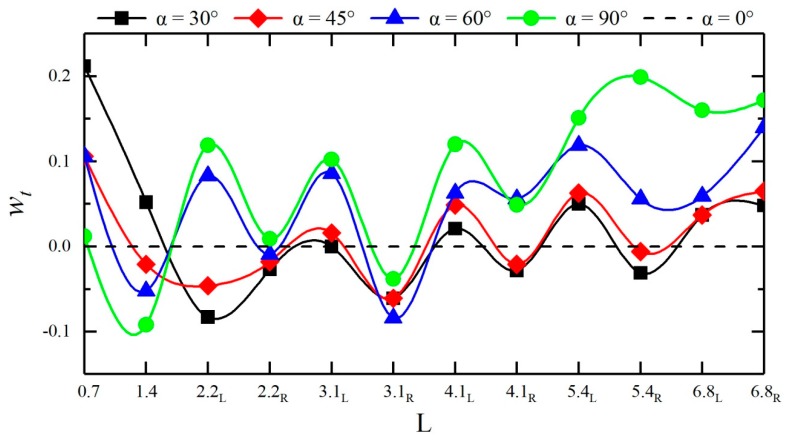
Rise time retardation factors *w_t_* at each measuring point of different confluence configurations.

Due to the confluence angle, the times for the dam-break flow to reach the left and right banks of each cross-section differ. As shown in Figure 11, the larger the confluence angle is, the sooner the dam-break flow reaches measuring points #4 and #5 in the confluence section, and the later the dam-break flow reaches other downstream measuring points; the times when the dam-break flow reaches the measuring points on the two sides of the same cross-section in the downstream straight section differ, basically following a pattern of reaching the right bank first and then the left bank. To systematically reflect the influences of different confluence angles on the routing of dam-break flow, a rise time retardation factor *w_t_* is introduced, which can be calculated using Equation (8):

(8) $$w_{t}=\frac{t_{j}-t_{s}}{t_{s}},$$

where *t_j_* is the rise time at a measuring point in the confluence channel and *t_s_* is the rise time at the same measuring point in the straight channel. A *w_t_* greater than 0 indicates that the confluence angle enhances the wave peak at this measuring point and vice versa. Figure 12 show the rise time retardation factors at each measuring point in the downstream straight sections of different configurations. The figure shows that under different confluence angles, the influences of the measuring points on the routing of the dam-break flood differ. When the confluence angle retards the routing of the dam-break flow, the times for the flow to arrive at different measuring points are delayed by approximately 0.91% to 21.18%. When the confluence angle accelerates the routing of the dam-break flow, the times for the flow to arrive at different measuring points are advanced by approximately 0.62% to 9.28%; under a confluence angle of 30°, the confluence section experiences the most remarkable retardation (retarded by approximately 5.15% to 21.18%). In the downstream straight section, the greater the confluence angle is, the more pronounced the retardation effect on flood is; when the confluence angle is 90°, the flood is retarded by 0.91% to 19.88%.

### 3.2. Pressure Variation

**Figure 13:**
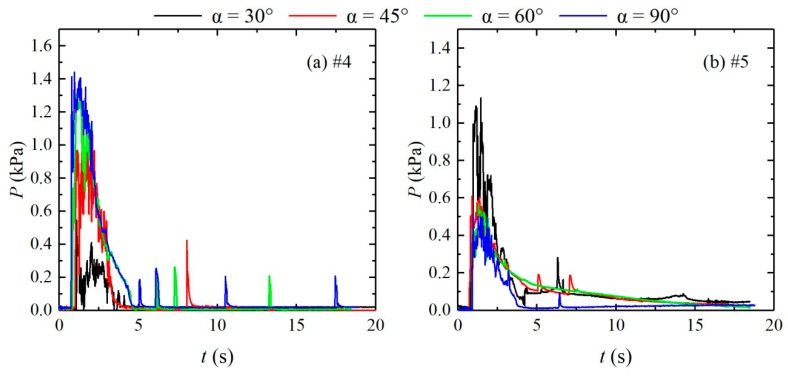
Pulsating pressure hydrographs at measuring points #4 and #5 of different configurations. (**a**): measuring points #4; (**b**): measuring points #5.

The instantaneous pressure hydrographs at measuring points #4 and #5 of different confluence angle configurations (Figure 13) and the peak pulsating pressures at each measuring point of different configurations (Table 3) are analyzed. The variations in the pulsating pressures at various measuring points are basically consistent with the variations of the water depth; however, after the pressure reaches the peak, the overall variation trends downward, which differs from the relatively large fluctuations in the water level time curves in the time period of 6–10 s, during which the pressure fluctuates relatively little. The flow reflected upward from the impact on the right bank of the confluence propagates again to the confluence, and the flow propagates downstream in a relatively straight manner; when the confluence angle gradually increases from 30° to 90°, the point of impact moves from measuring points #5 and #7 upstream to measuring points #3 and #4, and the peak of the pulsating pressure increases, that is, the impact intensity gradually increases with increasing confluence angle, which is consistent with the variation of the wave peak at the confluence section. The dam-break flow propagates downstream after impact and reflection at the confluence; when the confluence angle is 30°, the flow impacts measuring points #8 and #10 on the left bank and then moves toward measuring point #11; when the confluence angle is 45°, 60°, and 90°, the dam-break flow impacts measuring point #8 on the left bank and then moves toward measuring point #11; when the confluence angle is 90°, the dam-break flow impacts measuring points #6 and #8 on the left bank and then impacts measuring point #22 on the right bank. Therefore, under different confluence angles, the locations of the impact points of the dam-break flood on the two sides of the downstream straight section and the corresponding peak pressure differ, and the impact point moves upstream as the confluence angle increases.

### 3.3. Flow Field

**Figure 14:**
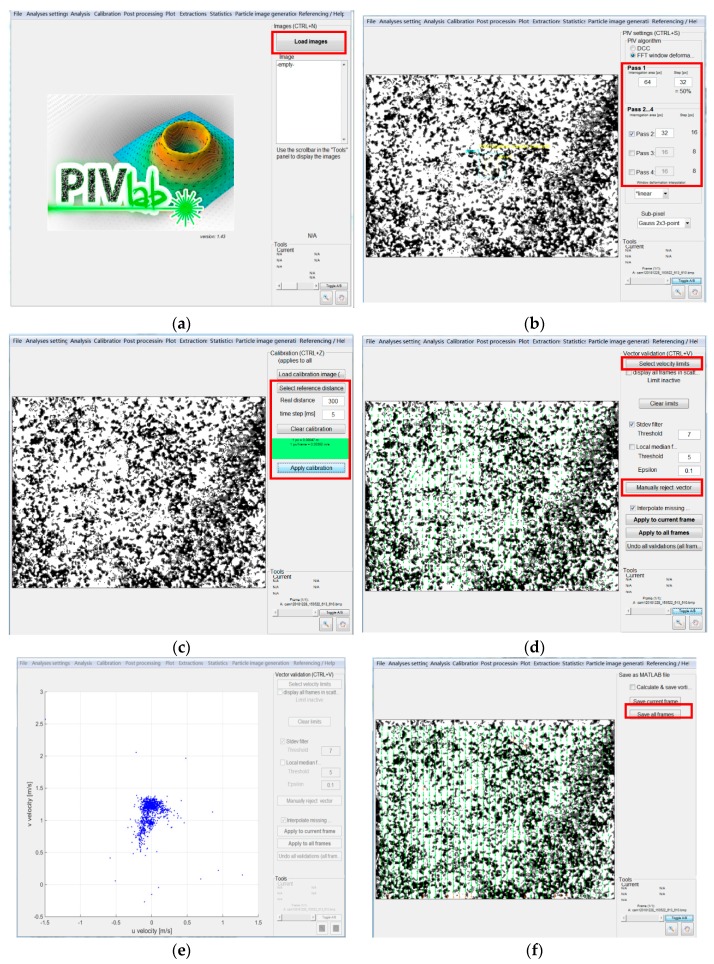
Procedure for particle image processing. (**a**) Image import; (**b**) window selection; (**c**) coordinate transformation; (**d**) local correction; (**e**) velocity range limit; (**f**) velocity vector output.

Figure 14 shows the procedure for particle image processing. The image is imported into the program for processing, as shown in Figure 14a. Figure 14b shows the window division during particle image processing. The acquired image is divided into numerous windows by the PIV technique, and the corresponding flow velocity vector information is obtained by analyzing the displacement of the particles within the window. As the images collected are in units of pixels, it is necessary to select a reference distance in the image and then match the actual distance in the experiment, thereby performing coordinate transformation, as shown in Figure 14c. After the velocity vector diagram is obtained, a flow velocity range is defined to exclude the flow velocity outliers. Alternatively, the outliers can be manually selected to correct the velocity vector diagram, as shown in Figure 14d,e. The corrected velocity vector information is output and then imported into Tecplot to plot the velocity vector diagram.

**Figure 15:**
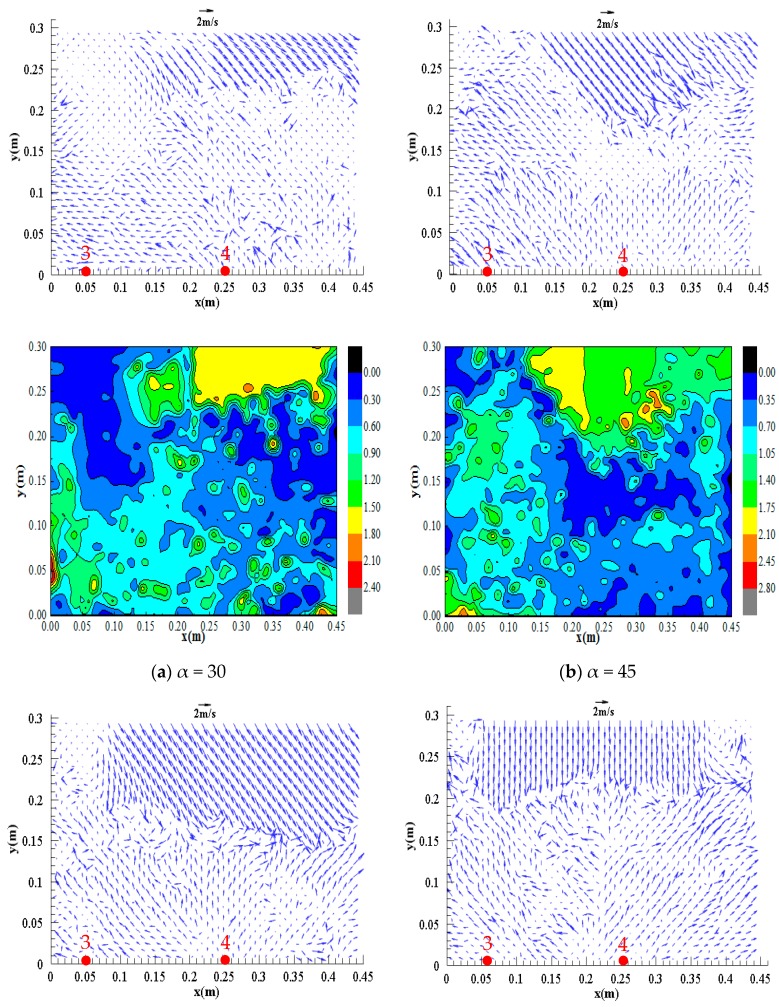

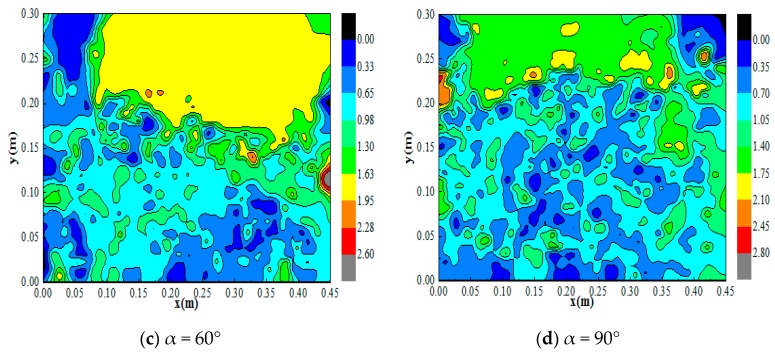
Distributions of the surface flow field of zone 1 under different configurations, (**a**) α = 30°; (**b**) α = 45°; (**c**) α = 60°; (**d**) α = 90°.

**Figure 16:**
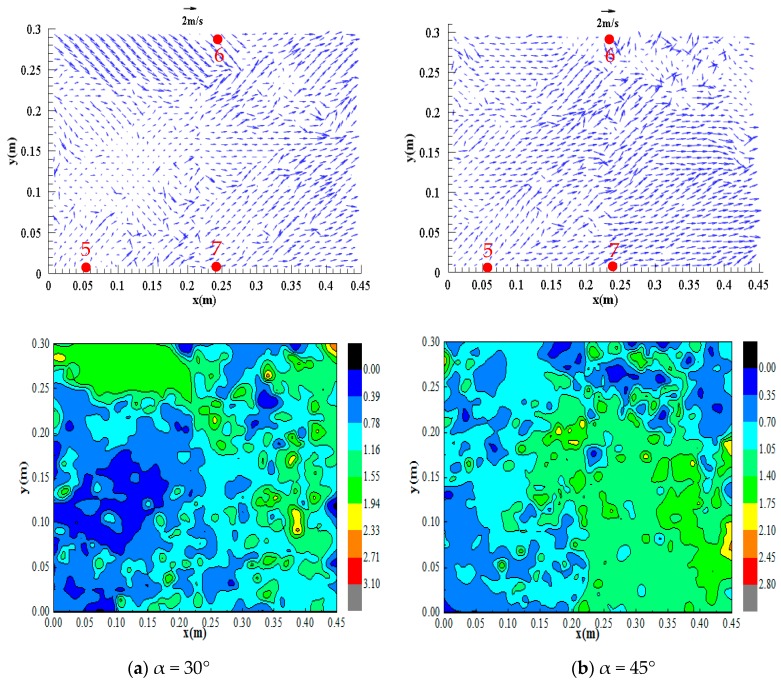

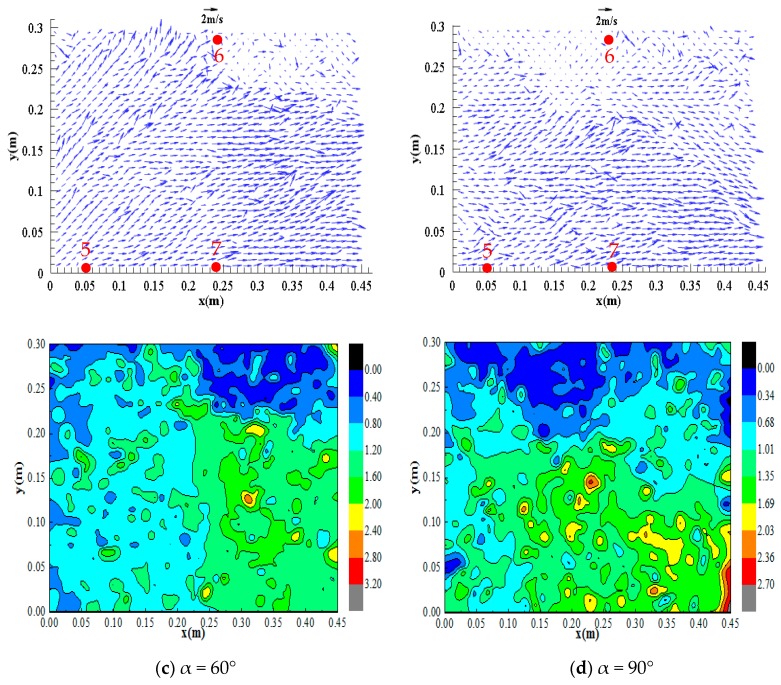
Distributions of the surface flow field of zone 2 under different configurations, (**a**) α = 30°; (**b**) α = 45°; (**c**) α = 60°; (**d**) α = 90°.

Figure 15 and Figure 16 show the distributions of the surface flow field of observation zones 1 (L = –0.17 to 1.23) and 2 (L = 1.23 to 2.63), respectively, in the confluence section with an initial upstream water depth of 0.30 m and different confluence configurations.

Figure 15 shows that the flow regime is evenly distributed in the inlet section, with flow velocities of approximately 1.50 –1.80 m/s. However, the flow regime at the location of impact on the right bank is relatively turbulent, and part of the flow is directed upstream and part of the flow is directed downstream at approximately 0.35–0.40 m/s, which is lower than the velocities in the inlet section. This phenomenon occurs because after the dam-break flow of the branch channel reaches the confluence section, the flow impacts the right bank of the confluence and generates a water level increase and reflected wave in the vicinity, resulting in a relatively low flow velocity and flow velocity in the opposite direction. Figure 16 shows that the flow velocities in zone 2 are all directed downstream; at confluence angles of 45°, 60°, and 90°, the reflected flow generated by dam-break flow impacting the right bank of the confluence section propagates to the downstream left bank and impacts the left bank again, resulting in a relatively low flow velocity of approximately 0.40 m/s in the vicinity.

Comparisons of the flow regimes and flow velocities in different zones with different confluence configurations reveals the following. At a confluence angle of 30°, the flow impacts measuring points #5, #7, and #8 with impacts ranging from L = 0.90 to 3.13; at a confluence angle of 45°, the flow impacts measuring points #4, #5, and #8 with impacts ranging from L = 0.50 to 2.97; at a confluence angle of 60°, the flow impacts measuring points #4, #6, and #8 with impacts ranging from L = 0.50 to 2.85; and at a confluence angle of 90°, the flow impacts measuring points #3, #4, and #6 with impacts ranging from L = 0 to 2.23. Therefore, as the confluence angle increases, the impact point moves upstream, and the impact range decreases.

### 3.4. Flow Discharge

As the flow discharges at different cross-sections could not be obtained in the physical experiment, we used the FLOW-3D software (Version 11.2, Los Alamos, NM, USA) to numerically simulate the variation in the downstream flow discharge. Comparison with the water levels and flow fields measured in the physical experiment shows that the results from numerical simulation are highly credible.

**Figure 17:**
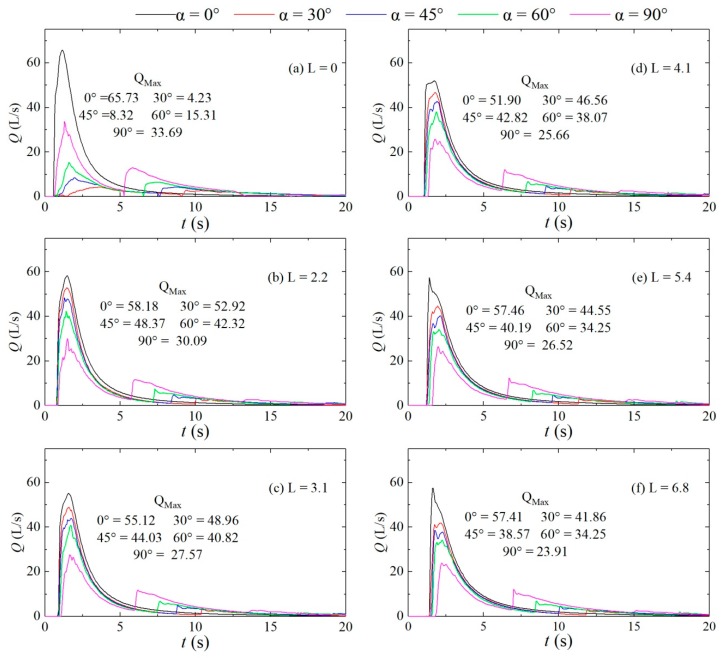
Flow discharge hydrographs for the downstream straight sections of different configurations. (**a**): L = 0; (**b**): L = 2.2; (**c**): L = 3.1; (**d**): L = 4.1; (**e**): L = 5.4; (**f**): L = 6.8.

Figure 17 shows the flow discharge hydrograph at each cross-section under different configurations. The figure shows that the variation of the flow discharge vs. time is basically the same as those of the water level and pressure vs. time. After reaching the peak, the right bank generates resistance to the flow due to the impact of the dam-break flow in the branch channel on the right bank of the confluence, thus forming the upstream and downstream reflected waves. Due to the returning of the upstream reflected wave, the flow discharge rises again from 5.5 to 12 s and then gradually decreases and stabilizes with time. The increase in the flow discharge is approximately 6.3% to 50.9% of the first peak flow discharge; at the same downstream cross-section location, the peak flood discharge in the confluence channel is lower than that in the straight channel, and the peak flood discharge decreases as the confluence angle increases. These phenomena occur because the larger the confluence angle is, the more significant the abatement effect on the peak flood discharge is. Additionally, at the downstream cross-sections, the time of rise in dam-break flow discharge, that is, the pre-peak arrival time, differs for different confluence angles: a larger confluence angle leads to a later initial arrival of the dam-break flow, a smaller peak flow discharge, and an earlier rise again along with a larger peak flow discharge and a higher rise rate, indicating that the larger the confluence angle is, the greater the obstruction to the flow is.

**Figure 18:**
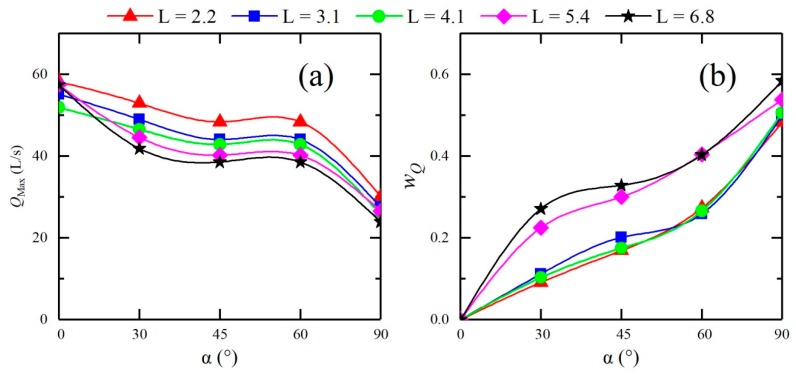
Peak flood discharge and discharge peak reduction factor at different cross-sections in the downstream straight sections of different configurations. (**a**): peak flood discharge; (**b**): discharge peak reduction factor.

The above analysis shows that the confluence has an abatement effect on the peak discharge of a dam-break flood in a confluent channel, and the flood peak reduction capability differs for different confluence angles. To more intuitively and easily demonstrate the capability of the confluence angle to reduce the peak flood discharge, the discharge reduction factor *w_Q_* is defined in Equation (9)

(9) $$w_{Q}=\left|\frac{Q_{j,max}-Q_{s,\max}}{Q_{s,\max}}\right|,$$

where *Q_j,max_* is the peak flood discharge at a cross-section of a confluent channel and *Q_s,max_* is the peak flood discharge at the same cross-section of a straight channel. Figure 18 shows the peak flood discharge and discharge reduction factor for each cross-section of the downstream straight sections of different configurations. Clearly, at the same cross-section, *w_Q_* increases as the confluence angle increases, that is, a larger confluence angle leads to a higher discharge reduction capability in the range of 9.05% to 58.36%.

## 4. Conclusions

This study investigated the influences of four different confluence angles (30°, 45°, 60°, and 90°) on the propagation of a dam-break flood. The flow and pressure variations at the confluence and in downstream cross-sections were obtained through physical model tests. The surface flow velocities were obtained using an image processing technique. In addition, Flow-3D software was used to obtain the flow variations at different downstream cross-sections. The results are as follows:

(1) The dam-break flow impacts the right bank at the confluence, causing the water level to rise and forming reflected waves moving upstream and downstream. The reflected waves moving upstream form backflow in the branch channel and 5–9 s later reach the confluence again; at this time, the flow moves gently along the channel and propagates straight downstream. However, the reflected waves moving downstream turn back during propagation in the downstream channel, showing remarkable asymmetry and resulting in water superelevation; the dam-break flood propagates at the confluence and in the downstream sections in a process of impact-reflection-return-attenuation.

(1) Compared with that of the straight channel, as the confluence angle increases, the maximum pressure at the confluence increases, the peak value point and flow impact point move upstream, and the impact point of the downstream straight section moves upstream.
(2) The flow regime is relatively turbulent at the confluence and is evenly distributed in the downstream straight section. Additionally, the flow velocity at the inlet of the confluence section is relatively high, and the flow velocity at the flow impact location is lower than that at the inlet and in the downstream straight section; as the confluence angle increases, the flood impact point moves upstream, and the impact range decreases.
(3) A wave peak factor *w_h_* was introduced to reveal the influence of the confluence angle on the peak of the downstream straight section. The confluence angle increases the wave peaks at the measuring points by approximately 0.19% to 99.49%, and the smaller the confluence angle is, the more significant the enhancement effect is. In comparison, when the confluence angle has an abatement effect on the wave peak values, the reduction is in the range of approximately 1.82% to 40.70%, and the larger the confluence angle is, the greater the abatement effect is.
(4) A rise time retardation factor *w_t_* and a discharge peak reduction factor *w_Q_* were introduced to reveal the influence of the confluence angle on the dam-break flood routing. The larger the confluence angle is, the more pronounced the retardation effect on the dam-break flood routing is, and the greater the abatement effect on the peak flood discharge is. Compared with that of the straight channel, the flood arrival time is delayed by approximately 0.91% to 21.18%, and the flow discharge is reduced by approximately 9.05% to 58.36%.

Due to the lack of detailed experimental study in the literature on the dam-break flood routing in confluent channels, the current laboratory data can provide reference for the verification of numerical solutions of the scientific community. In the future, to better understand confluent channels regarding the dam-break flow propagation impact, follow-up research can consider the river section shape, roughness, channel slope, and other factors for in-depth study.

## Figures and Tables

**Table 1:** Different test conditions.

Upstream Water Level	Confluence Angles				
	30°	45°	60°	90°	0° (Straight)
0.30 (m)	#1	#2	#3	#4	#5
0.40 (m)	#6	#7	#8	#9	#10
0.45 (m)	#11	#12	#13	#14	#15

**Table 2:** Cross-sections and locations for flow measurements.

**Baffles**	1	2	3	4	5	6
**L**	0	2.2	3.1	4.1	5.4	6.8

**Table 3:** Peak pulsating pressures at different measuring points of different configurations.

Measuring Points	Confluence Angles			
	30°	45°	60°	90°
#3	0.002	0.303	0.448	0.733
#4	0.546	1.154	1.321	1.443
#5	1.134	0.611	0.569	0.511
#6	0.510	0.413	0.550	0.652
#7	1.010	0.856	0.640	0.565
#8	0.577	0.486	0.729	0.451
#9	0.543	0.304	0.390	0.153
#10	0.661	0.362	0.598	0.114
#11	0.257	0.652	0.614	0.336
